# Integrin αvβ6 plays a bi-directional regulation role between colon cancer cells and cancer-associated fibroblasts

**DOI:** 10.1042/BSR20180243

**Published:** 2018-12-07

**Authors:** Cheng Peng, Xueqing Zou, Wanying Xia, Huijie Gao, Zequn Li, Naiqing Liu, Zongquan Xu, Chao Gao, Zhaobin He, Weibo Niu, Ruliang Fang, Siddhartha Biswas, Michael Agrez, Xuting Zhi, Jun Niu

**Affiliations:** 1 Department of General Surgery, QiLu Hospital, Shandong University, Jinan, Shandong, China; 2 The Institute of Laparoscopic Minimally Invasive Surgery of Shandong University, Shandong University, Jinan, Shandong, China; 3 Department of General Surgery, Linyi Central Hospital, Linyi, Shandong, China; 4 Department of General Surgery, Jiangxi Provincial Tumor Hospital, Nanchang, Jiangxi, China; 5 Newcastle Bowel Cancer Research Collaborative, The University of Newcastle, Callaghan, New South Wales, Australia

**Keywords:** Colon Cancer, Cancer associated fibroblasts, Integrin αvβ6, SDF-1, TGF-β

## Abstract

Tumor microenvironment (TME) is the cellular environment in which tumor exists, and it contributes to tumor formation and progression. The TME is composed of tumor cells, stromal cells, cytokines, and chemotactic factors of which fibroblasts are the main cellular components. In our present study, we found that colorectal cancer (CRC) cells expressing integrin αvβ6 clearly could induce morphological changes in inactive fibroblasts and increased the expression of activated fibroblast markers such as α-smooth muscle actin (α-SMA) and fibroblast-activating protein (FAP). Those activated fibroblasts in the TME are called cancer-associated fibroblasts (CAFs). In order to investigate the mechanism by which CRC cells expressing integrin αvβ6 activated CAFs, a series of assays have been carried out in the follow-up. We found that CRC cells could secrete inactive transforming growth factor β (TGF-β); however, integrin αvβ6 activated TGF-β, which subsequently activated fibroblasts. This process was disrupted by knockdown of integrin αvβ6. In contrast, activated fibroblasts could promote CRC cell invasion. In particular, the strengthening effect on expression of integrin αvβ6 in colon cancer cells was obvious. Additionally, we found that CAFs could secrete stromal cell-derived factor-1 (SDF-1) and promote CRC cell metastasis in distant organs via the SDF-1/C–X–C chemokine receptor type 4 (CXCR4) axis. Taken together, we assumed that CRC cells and CAFs activated one another and worked together to promote cancer progression, with integrin αvβ6 playing a role in the bi-directional regulation of these cells. Hence, integrin αvβ6 may serve as a therapeutic target for the future CRC treatment.

## Introduction

Almost 130 years ago, Paget [1] put forth the famous ‘seed and soil’ theory to explain the metastatic preference of cancer cells for specific organs. The cancer cells were considered the ‘seeds’, and the specific organ microenvironment was the ‘soil’; interactions between the ‘seeds’ and ‘soil’ were proposed to determine the formation of a secondary tumor. With subsequent research, the concept of the tumor microenvironment (TME) gradually gained empirical support [2,3]. The TME is composed of many types of cells and proteins, such as fibroblasts, inflammatory cells, immune cells, vascular endothelial cells, mesenchymal stem cells (MSCs), and extracellular macromolecules including collagen, laminin, and protein polysaccharides. The TME does not only function as a scaffold to support tissue architecture and integrity but also secretes cytokines, growth factors, and protease.

Fibroblasts are the main cellular components of the TME; they synthesize collagen and other extracellular matrix (ECM) proteins, which provide the structural framework (stroma) for animal tissues. Under normal conditions, fibroblasts are inactive (fibrocytes) in the resting state and are involved in maintenance and tissue metabolism [4]. They are activated when tissues are injured, and play a role in processes such as wound healing and inflammation. Usually, fibroblasts not only synthesize components of the ECM but also secrete proteases. When they are recruited to the TME, cells within the tumor transform the surrounding stromal fibroblasts into cancer-associated fibroblasts (CAFs) [5]. CAFs differ from normal fibroblasts (NFs) in appearance and function [6]. They can survive in the TME for a fairly long period of time, and do not undergo normal apoptosis [7]. CAFs are derived from several cell types. When activated by cells in the surrounding stroma, CAFs can secrete many types of growth factors, cytokines, and proteases, which promote tumor cell proliferation, invasion, and angiogenesis [8]. Activated CAFs express specific proteins, such as α-smooth muscle actin (α-SMA) and fibroblast-activating protein (FAP) [9].

Our previous study showed that αvβ6 is a special integrin subtype that is only expressed in epithelial cells; its major ligand is fibronectin. In normal epithelial cells, αvβ6 can hardly be detected [10], but its expression significantly increases in response to injury and/or inflammation as well as in epithelial tumors, such as those occurring in gastric and colon cancers (colorectal cancers, CRCs) [11–13]. As a cell adhesion molecule, integrin αvβ6 mediates cell–cell and cell–ECM events through binding ECM proteins [14]. Integrin αvβ6 also participates in the epithelial–mesenchymal transition (EMT) of CRC cells, and the αvβ6-ERK-Ets1 signaling pathway plays an important role in this process [15,16].

In the present study, the human CRC cell lines HT-29 and RKO were used to investigate whether integrin αvβ6 mediates the relationship between cancer cells and CAFs, and to determine the underlying mechanisms.

## Materials and methods

### Cell lines and culture conditions

The human normal colonic fibroblast cell line CCD-18Co and human CRC cell lines HT-29, RKO, Caco-2, SW480, LoVo, and SW620 were obtained from the American Type Culture Collection (Gaithersburg, MD, U.S.A.). Stable transfectants of RKO cells expressing pcDNA1neo constructs containing wild-type β6 or HT-29 cells expressing β6 siRNA were prepared as previously reported [16]. Normal human colonic fibroblast cell line CCD-18Co were cultured in fibroblast medium supplemented with 2% FBS (Sigma, St. Louis, MO, U.S.A.), 1% fibroblast growth supplement, and 1% penicillin/streptomycin solution. The CRC cell lines were maintained as monolayers in standard Dulbecco’s modified Eagle’s medium (4.5 g/l glucose; Sigma) with 10% heat-inactivated FBS, 20 mM HEPES, 100 IU/ml penicillin, and 100 μg/ml streptomycin (Merck, Darmstadt, Germany). The cells were incubated at 37°C with 5% CO_2_ and saturated humidity. Cells were subcultured until they were more than 90% confluent. Cells at passages 3–6 were used for experiments.

Indirect co-culture of fibroblasts with cancer cells were used in the study to activate the fibroblast as previously described and modified based on the preliminary experiments [17,18]. CCD-18Co cells (3.0 × 10^5^) were seeded in six-well culture plate. Colon cancer cells (3.0 × 10^5^) were seeded in a 0.4-μm polyester membrane of a 24-mm transwell insert and placed in a separate culture plate. The next day, the transwell insert was transferred to the culture plate containing fibroblast cells, and co-cultured for 96 h. Co-cultured cells were cultured in DMEM/F12 medium (10% FBS), change medium every 48 h, and were incubated at 37°C with 5% CO_2_ and saturated humidity. In some situation, fibroblast cells were seeded in the insert while colon cancer cells were cultured in the well according to the purpose of the experiments.

### Antibodies and reagents

The mouse-anti-human monoclonal antibody R6G9 (IgG_2a_), which is directed against the extracellular domain of human integrin subunit β6, was obtained from Chemicon International (Temecula, CA, U.S.A.). The following monoclonal antibodies were obtained from Abcam (Cambridge, MA, U.S.A.): ab5694 and ab28244, which target the CAF markers α-SMA and FAP, respectively: ab27969, which targets transforming growth factor β (TGF-β); and ab9797, ab9695, ab16828, and ab6672, which target the four cytokines stromal cell-derived factor-1 (SDF-1), epidermal growth factor (EGF), basic fibroblast growth factor (bFGF), and interleukin 6 (IL-6). EP1186Y and EP1254 monoclonal antibodies, which target matrix metalloproteinase (MMP) 3 (MMP-3) and MMP-9, respectively, were also purchased from Abcam. Reagents for SDS/PAGE and molecular weight markers were obtained from Bio-Rad Laboratories (Hercules, CA, U.S.A.). The C–X–C chemokine receptor type 4 axis (CXCR4) antagonist AMD3100 was purchased from Santa Cruz Biotechnology (Santa Cruz, CA, U.S.A.).

### RNA extraction and RT-PCR analysis

Total cellular RNA was extracted using TRIzol reagent (Sigma), and cDNA was synthesized according to the manufacturer’s instructions (Promega, Madison, WI, U.S.A.). Equal amounts of cDNA were subjected to PCR analysis. The primer sequences were as follows: β6 integrin forward: 5′-AGGATAGTTCTGTTTCCTGC-3′ and β6 integrin reverse: 5′-ATCATAGGAATATTTGGAGG-3′, which generated a 141-bp amplicon; α-SMA forward: 5′-CGTGGCTACTCCTTCGTG-3′ and α-SMA reverse: 5′-TGATGACCTGCCCGTCT-3′,which generated a 160-bp amplicon; FAP forward: 5′-TCAACTGTGATGGCAAGAGCA-3′ and FAP reverse: 5′-TAGGAAGTGGGTCATGTGGGT-3′, which generated a 219-bp amplicon; GAPDH forward: 5′-GTCAGTGGTGGACCTGACCT-3′ and GAPDH reverse: 5′-TGAGGAGGG GAGATTCAGTG-3′, which generated a 400-bp amplicon and was used as the internal control. The amplification conditions were 33 cycles at 94°C for 30 s, 51°C for 30 s, and 72°C for 30 s. The PCR products were electrophoresed on a 1.5% agarose gel and detected by Ethidium Bromide staining. Data were analyzed with AlphaImager software (Alpha Innotech Co., San Leandro, CA, U.S.A.).

### Western blotting

CCD-18Co cells were co-cultured with various CRC cells for 96 h. Both adherent and floating cells were collected and frozen at −80°C. To detect the levels of potentially mechanistic proteins, these proteins were extracted from the cells for the determination of concentration. The protein concentration was measured using the BCA Protein Assay Reagent, and equal amounts of protein (10 μg) were loaded on to a 12.5% SDS/PAGE gel and electrophoresed under non-reducing conditions. After electrophoresis, the proteins were transferred to nitrocellulose membranes. Equivalent protein loading in each lane was confirmed by staining the nitrocellulose membranes with Ponceau-S Stain using the 42-kDa β-actin band as a reference marker. Then, the membranes were probed with primary monoclonal antibodies against key signaling pathway factors followed by peroxidase–conjugated secondary antibodies. Proteins on the membranes were visualized using the Enhanced Chemiluminescence Detection System (Amersham, Aylesbury, U.K.) according to the manufacturer’s instructions. Optical density was analyzed with ImageJ software.

### MMP activity assay

MMP-3 and MMP-9 levels in the conditioned medium obtained from co-cultured cells were assayed using the commercially available kit Biotrak MMP Activity Assay System (Amersham, Aylesbury, U.K.). This assay measured total MMP levels (inactive pro-enzyme artificially activated and endogenous active enzyme), and MMP secretion was calculated on a per-cell basis.

### Enzyme-linked immunoassay

The levels of TGF-β1 and SDF-1 were detected using the appropriate enzyme-linked immunoassay (ELISA) kit (R&D systems, Minneapolis, MN, U.S.A.) according to the manufacturer’s instructions.

### Transwell assay for cell invasion

The invasive ability of the CRC cells was analyzed in 24-well Boyden chambers with polycarbonate membranes (8-μm pore size; CoStar, Boston, MA, U.S.A.). The membranes were pre-coated with 50 μl Matrigel (BD Biosciences, San Diego, CA, U.S.A.) to form matrix barriers. Colon cancer cells were re-suspended in 100 μl serum-free medium at a concentration of 1 × 10^6^ cells/ml, and added to the upper chamber; 1 × 10^5^ fibroblast cells were seeded in the lower compartments with 600 μl medium containing 10% FBS. After incubation, the cells remaining on the upper surface of the membrane were removed. Cells on the lower surface of the membrane were fixed and stained with Crystal Violet, and counted under a light microscope at 200× magnification.

### Statistical analysis

The results were expressed as the mean ± S.D. Comparisons between the two groups were performed with the Student’s *t*test. Comparisons of multiple samples and rates were performed using the single-factor ANOVA. All statistical analyses were performed using SPSS for Windows version 16.0. *P*-values less than 0.05 were considered statistically significant.

## Results

### Integrin αvβ6 is expressed in CRC cell lines and promotes the activation of fibroblasts

**Figure 1:**
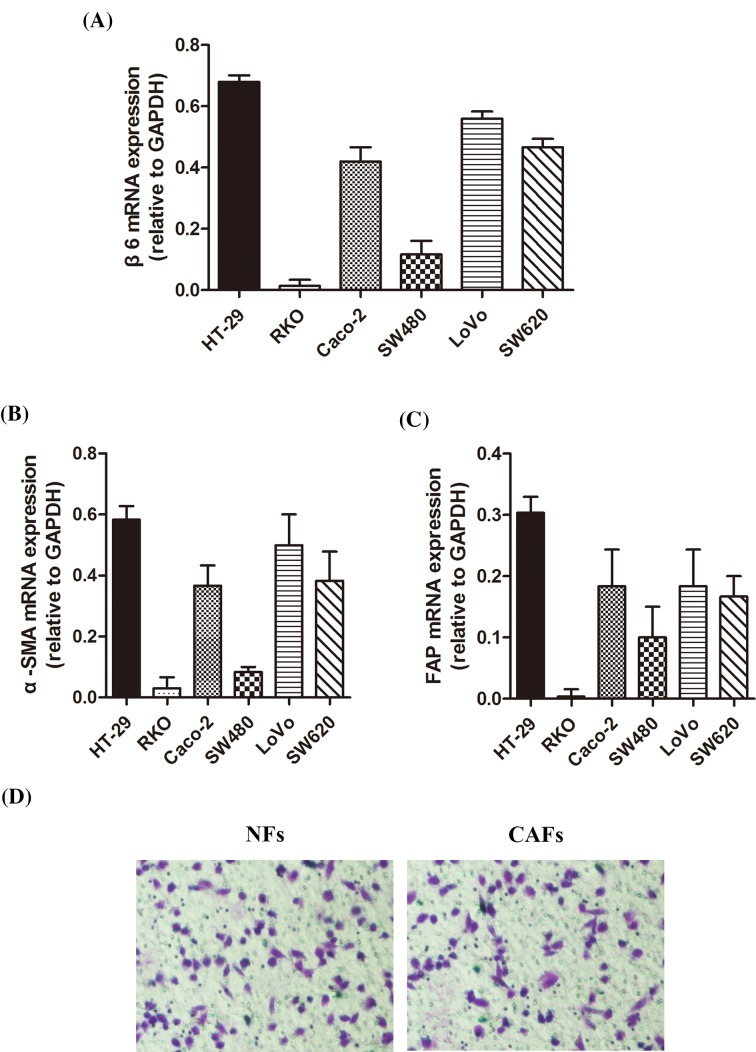
Integrin αvβ6 is expressed in CRC cell lines and promotes the activation of fibroblasts (**A**) RT-PCR assay shows *β6* mRNA expression in six types of CRC cell lines. (**B**) RT-PCR assay shows *α-SMA* mRNA expression in the media collected from CCD-18Co cells co-cultured with the above-mentioned CRC cell lines. (**C**) RT-PCR assay shows *FAP* mRNA expression in the media collected from CCD-18Co cells co-cultured with the above-mentioned CRC cell lines. (**D**) Invasion experiment shows no difference observed between CAFs activated by cancer cells and those without cancer cells pretreatment. Data are mean ± S.E.M. from three independent experiments.

To investigate the expression of β6 in CRC cell lines, we selected six types of CRC cell lines and performed RT-PCR to detect *β6* mRNA levels. The results showed that *β6* mRNA expression was high in HT-29, Caco-2, Lovo, and SW620 CRC cell lines, with the highest expression observed in HT-29 cells and the lowest expression found in RKO cells (Figure 1A). To investigate the effects of these CRC cells on CCD-18Co fibroblasts, we co-cultured them with CCD-18Co fibroblasts for 96 h. Next, we performed RT-PCR to detect the mRNA levels of α-SMA and FAP. The results of these assays showed that *α-SMA* and *FAP* mRNA levels in CCD-18Co fibroblasts varied according to the type of CRC cell line. The mRNA level of α-SMA was tightly correlated with β6 expression and exhibited the same expression pattern, as shown in Figure 1B. Similar results were observed with *FAP* mRNA expression (Figure 1C).

To avoid the individual difference between NFs and CAFs used in the study effecting the result of transwell experiments, invasion experiment was done with CAFs activated by cancer cells and those without cancer cellls pretreatment. There was no difference observed between NFs and CAFs (Figure 1D).

### Regulation of integrin αvβ6 expression in CRC cells can affect fibroblast activation

**Figure 2:**
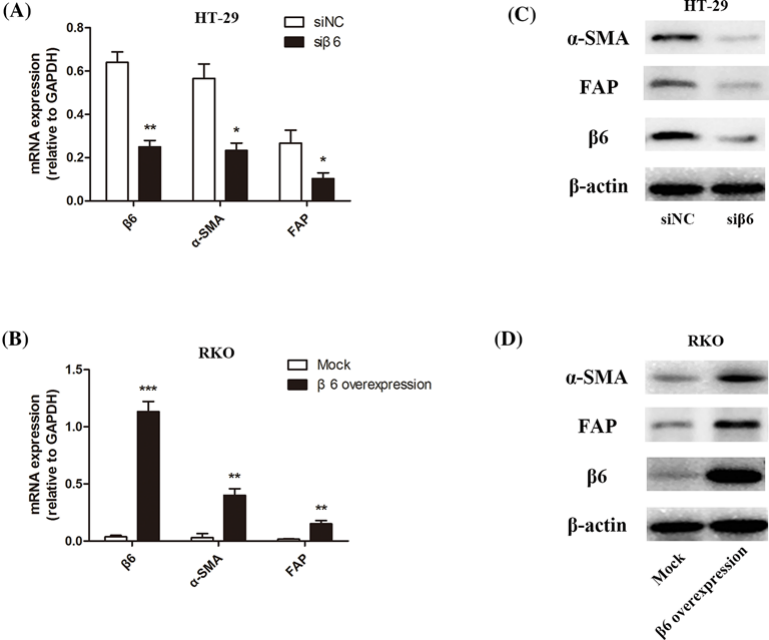
Regulation of integrin αvβ6 expression in CRC cells can affect fibroblast activation (**A**) RT-PCR assay shows the *β6, α-SMA*, and *FAP* mRNA levels in β6 expressing siRNA negative control HT-29 cells (siNC) and siRNA targetting β6 expression HT-29 cells (siβ6). In accordance with the decrease in β6 expression between siNC and siβ6 (**P<0.01), the mRNA expression of α-SMA and FAP decreased significantly (*P<0.05). (**B**) RT-PCR assay shows the *β6, α-SMA*, and *FAP* mRNA levels in mock transfected (Mock) RKO CRC cells and β6 transfected (β6 overexpression) RKO CRC cells. In accordance with the increase in β6 expression between Mock and β6 overexpression (***P<0.001), the mRNA expression of α-SMA and FAP significantly increased (**P<0.01). (**C**) Western blot analysis shows β6, α-SMA, and FAP protein levels in β6 expressing siRNA negative control HT-29 cells (siNC) and siRNA targetting β6 expression HT-29 cells (siβ6). The decreased expression of β6 was accompanied by a significant decrease in α-SMA and FAP protein expression. (**D**) Western blot analysis shows β6, α-SMA, and FAP protein expression in mock transfected (Mock) RKO CRC cells and β6 transfected (β6 overexpression) RKO CRC cells. The protein expression of α-SMA and FAP increased significantly with an increase in β6 expression. ***P<0.001, **P<0.01, *P<0.05; data are mean ± S.E.M. from three independent experiments.

To investigate the relationship between β6 expression in CRC cells with the fibroblast markers α-SMA and FAP, we selected HT-29 and RKO cells, which had the highest and lowest expression levels of β6, respectively. We established β6 knockdown HT-29 cells (siβ6) via siRNA technology and β6 overexpressing RKO cells (β6 overexpression) via plasmid transfection. Meanwhile, we also established β6 siRNA negative control HT-29 cells (siNC) and mock plasmid transfection RKO cells (Mock). Then CCD-18Co fibroblasts were co-cultured with these CRC cells for 96 h, followed by RT-PCR and Western blotting to detect the mRNA and protein expression of α-SMA and FAP, respectively, in the fibroblasts. In β6 knockdown cells, the decreased expression of β6 was accompanied by a significant decrease in *α-SMA* and *FAP* mRNA expression in CCD-18Co fibroblasts (*P<0.05 and **P<0.01, respectively). Accordingly, the overexpression of β6 in RKO CRC cells resulted in a significant increase in *α-SMA* and *FAP* mRNA expression in CCD-18Co fibroblasts (**P<0.01 and ***P<0.001, respectively), as shown in Figure 2A,B. The same expression patterns of α-SMA and FAP were observed at the protein level (Figure 2C,D).

### αvβ6 activates latent TGF-β, which induces fibroblast activation

#### αvβ6 activates latent TGF-β

**Figure 3:**
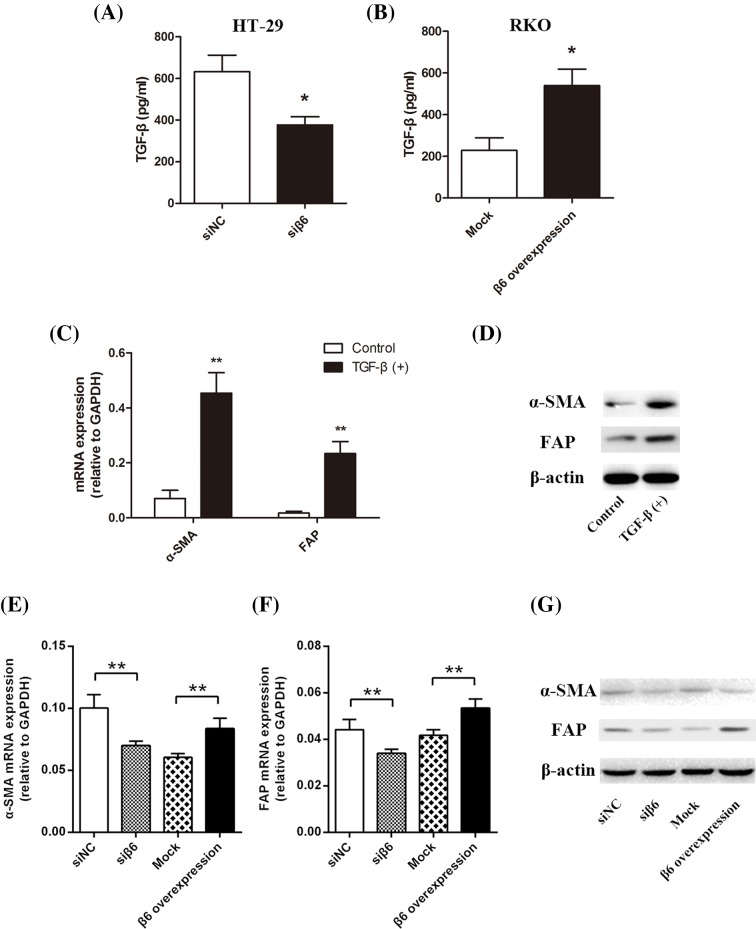
αvβ6 activates latent TGF-β (**A**) ELISA shows TGF-β levels in culture media collected from β6 expressing siRNA negative control HT-29 cells (siNC) and siRNA targetting β6 expression HT-29 cells (siβ6). Accompanying the decreased expression of β6, TGF-β level decreased significantly (*P<0.05). (**B**) ELISA show TGF-β levels in the culture media collected from mock transfected (Mock) RKO CRC cells and β6 transfected (β6 overexpression) RKO CRC cells. Accompanying the increased expression of β6, TGF-β levels increased significantly (*P<0.05). (**C**) RT-PCR assay shows *α-SMA* and *FAP* mRNA levels in inactive CCD-18Co fibroblasts. Accompanying the addition of exogenous *TGF-β, α-SMA*, and *FAP* mRNA levels significantly increased (**P<0.01). (**D**) Western blot analysis shows α-SMA and FAP protein levels in inactive CCD-18Co fibroblasts. Accompanying the addition of exogenous TGF-β, α-SMA, and FAP protein levels increased significantly. (**E**) RT-PCR assay shows mRNA α-SMA level in CCD-18Co fibroblasts co-cultured for 96 h with the above-mentioned four CRC cell lines. The expression of β6 was positively correlated with *α-SMA* mRNA levels in CCD-18Co cells. (**F**) RT-PCR assays show the *α-SMA* mRNA levels in fibroblasts CCD-18Co co-cultured for 96 h with the above-mentioned four CRC cell lines. The expression of β6 was positively correlated with *FAP* mRNA levels in the CCD-18Co cells. (**G**) Western blot analysis shows α-SMA and FAP protein levels in CCD-18Co cells co-cultured for 96 h with the above-mentioned four CRC cell lines. The expression of β6 was positively correlated with α-SMA and FAP protein expression in the CCD-18Co cells. **P<0.01, *P<0.05; data are mean ± S.E.M. from three independent experiments.

TGF-β is secreted by many cell types in a latent form in which it is complexed with two other polypeptides: latent TGF-β-binding protein and latency-associated peptide (LAP), a protein derived from the N-terminal region of the *TGF-β* gene product. To determine if TGF-β can be activated by integrin αvβ6, we incubated 10 μg TGF-β-LAP with the CRC cell lines for 24 h. Then, we collected the cell culture media for use in ELISA. We found that active TGF-β levels significantly decreased in β6 HT-29 knockdown cells (*P<0.05) but increased in RKO cells overexpressing β6 plasmid (*P<0.05), as shown in Figure 3A,B, respectively.

#### TGF-β induces fibroblast activation

We treated CCD-18Co fibroblasts with 10 μg/ml TGF-β1 for 24 h, and subsequently performed RT-PCR and Western blotting to detect the mRNA and protein levels, of α-SMA and FAP, respectively. The dose of TGF-β was based on previous studies and our preliminary experiments [19–21]. Treatment with TGF-β1 significantly increased the mRNA and protein levels of α-SMA and FAP (**P<0.01), as shown in Figure 3C,D, respectively.

#### Integrin αvβ6 promotes the activation of fibroblasts

To investigate the role of integrin αvβ6 in regulating active fibroblasts, we co-cultured CCD-18Co fibroblasts with the above-mentioned four types of CRC cells for 96 h, and followed by RT-PCR and Western blotting to detect the mRNA and protein expression, respectively, of α-SMA and FAP in fibroblasts. The results showed a positive correlation between β6 expression in CRC cells, and the mRNA levels of α-SMA and FAP in CCD-18Co fibroblasts, as shown in Figure 3E,F. This correlation was also observed at the protein level (Figure 3G).

### Integrin αvβ6 and CAFs induce CRC invasion

#### Integrin αvβ6 and CAFs induce CRC cell invasion

**Figure 4:**
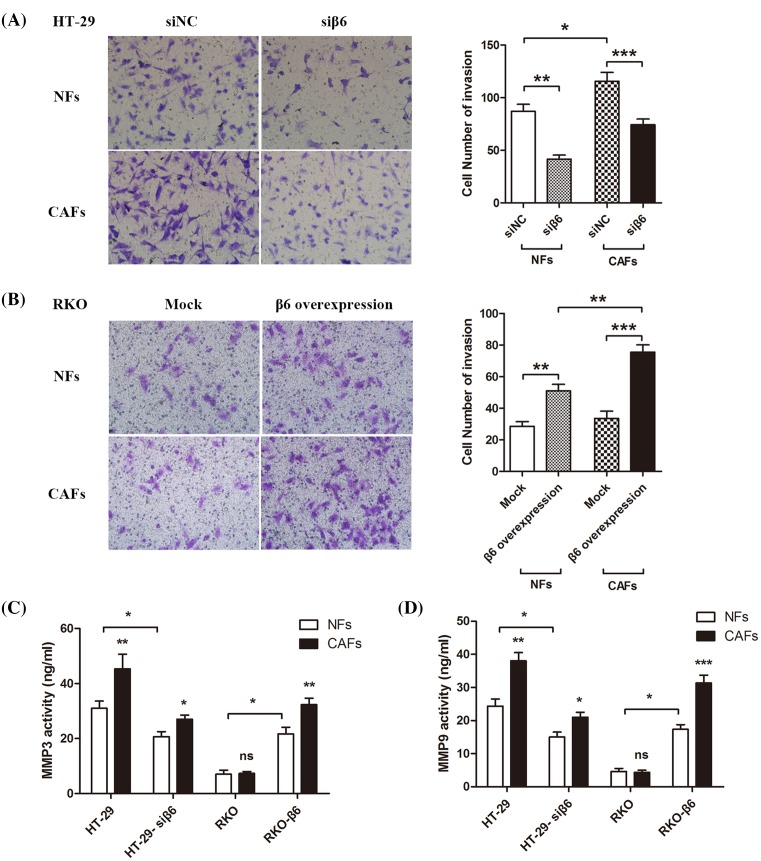
Integrin αvβ6 and CAFs induce CRC invasion (**A**) Transwell invasion chamber assay shows the invasive ability of siRNA negative control (siNC) and β6 knockdown HT-29 colon cancer cells (siβ6) after being co-cultured with NFs or CAFs for 24 h. The invasion capability of the CRC cells was positively correlated with β6 expression. Upon co-culture with NFs, the number of invasive cells significantly decreased in accordance with β6 knockdown in HT-29 cells (**P<0.01). This effect was also observed in CAF co-cultures (***P<0.001). Compared with the NFs, CAFs clearly promoted invasion of the negative control HT-29 cells (*P<0.05). (**B**) Transwell invasion chamber assay shows the invasive capability of mock transfected (Mock) and β6 transfected RKO CRC cells (β6 overexpression) after being co-cultured with NFs or CAFs for 24 h. The invasive capacity of CRC cells was positively correlated with β6 expression. Upon co-culture with NFs, the number of invasive cells significantly increased in accordance with the overexpression of β6 in RKO cells (**P<0.01). This effect was also in CAF co-cultures (***P<0.001). Compared with the NFs, CAFs clearly promoted the invasion of RKO cells overexpressing β6 (**P<0.01). (**C**) MMP3 ELISA shows that the four above-mentioned CRCs secreted MMP-3 after co-culture with NFs or CAFs for 24 h. CAFs promoted secretion of MMP-3 from CRC cells, and integrin αvβ6 increased the levels of secreted MMP-3 (*P<0.05, **P<0.01). (**D**) MMP-9 ELISA shows that the four above-mentioned CRC cells secreted MMP-9 after co-culture with NFs or CAFs for 24 h. CAFs promoted MMP-9 secretion from CRC cells, and integrin αvβ6 increased the levels of secreted MMP9 (*P<0.05,**P<0.01,***P<0.001). ***P<0.001, **P<0.01, *P<0.05; data are mean ± S.E.M. from three independent experiments.

To investigate the role of integrin αvβ6 and active CAFs in regulating cancer invasion, we incubated the co-culture of active fibroblasts CCD-18Co (CAFs) or NFs with the above-mentioned four types of CRC cells in upper and lower chamber of the transwell migration assay system for 24 h. Then, we observed the cell morphology and counted the number of cells in the lower chamber. The results showed that in HT-29 colon cancer cells and the siβ6 knockdown group, integrin αvβ6 promoted CRC cell invasion in the absence or presence of co-cultured CAFs, and the number of the invasive cancer cells significantly increased (**P<0.01 and ***P<0.001, respectively). Likewise, CAFs induced cell invasion when co-cultured with CRC cells with high or low β6 expression (*P<0.05), as shown in Figure 4A. These results were confirmed in RKO CRC cells and the β6 overexpression group (*P<0.05, **P<0.01, ***P<0.001, respectively), as shown in Figure 4B.

#### Integrin αvβ6 and CAFs promote MMP secretion

To further investigate the role of integrin αvβ6 and active CAFs in the regulation of cancer cell invasion, we co-cultured active fibroblasts CCD-18Co (CAFs) or NFs with the above-mentioned four types of CRC cells for 24 h. Then, we collected the cell culture media and performed MMP ELISA. Integrin αvβ6 induced secretion of MMP-3 in the absence or presence of co-cultured CAFs (*P<0.05); meanwhile, the CAFs also significantly induced MMP-3 secretion from CRC cells in the presence (**P<0.01) or absence (*P<0.05) of β6 expression, as shown in Figure 4C. Integrin αvβ6 also induced secretion of MMP-9 in the absence or presence of co-cultured with CAFs (*P<0.05); the CAFs also significantly induced MMP-9 secretion from HT-29 CRC cells and RKO β6 overexpression CRC cells (**P<0.01 and ***P<0.001, respectively). This type of induction effect was even found in siβ6 CRC cells (*P<0.05), as shown in Figure 4D.

### SDF-1 secreted by CAFs via the SDF-1/CXCR4 axis promotes CRC cell metastasis

#### CAFs secrete multiple cytokines

**Figure 5:**
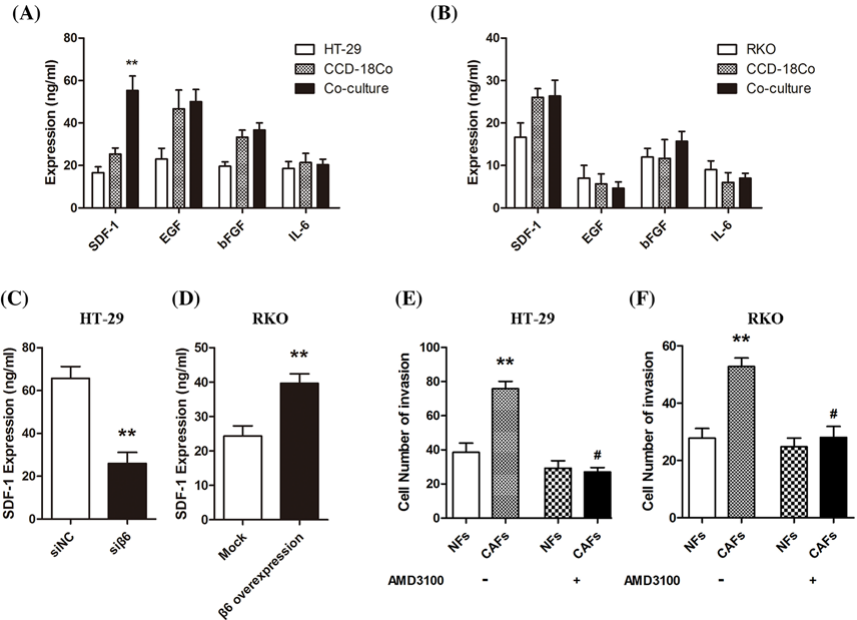
CAFs secrete multiple cytokines (**A**) ELISA shows cytokine levels in culture media collected from the HT-29 CRC cells and CCD-18Co fibroblasts co-cultured for 96 h. Only SDF-1 increased significantly in the co-culture medium (**P<0.01); there were no statistically significant changes in the levels of EGF, bFGF, and IL-6. (**B**) ELISA shows cytokine levels in the culture media collected from the RKO CRC cells and CCD-18Co fibroblasts co-cultured for 96 h. There were no statistically significant changes in the four cytokines examined. (**C**) ELISA shows SDF-1 expression in culture media collected from the siRNA negative control (siNC) and β6 knockdown HT-29 CRC cells (siβ6) after co-culture with CAFs for 96 h. Accompanying the decreased expression of β6, SDF-1 expression decreased significantly (**P<0.01). (**D**) ELISA shows SDF-1 expression in the culture media collected from mock transfected (Mock) and β6 transfected RKO CRC cells (β6 overexpression) after co-culture with CAFs for 96 h. Accompanying the increased expression of β6, SDF-1 expression increased significantly (**P<0.01). (**E,F**) Transwell invasion chamber assay shows the invasive capacity of HT-29 and RKO CRC cells after co-culture with NFs or CAFs for 24 h in the absence or presence of AMD3100 (a CXCR4 antagonist) pretreatment. The CRC cell invasion capability increased significantly when co-cultured with CAFs (**P<0.01); however, this effect was inhibited by AMD3100 pretreatment. **P<0.01, #*P*<0.05; data are mean ± S.E.M. from three independent experiments.

To investigate whether the cytokines secreted by CAFs are responsible for the progression of CRC cells, we co-cultured the CCD-18Co fibroblasts with CRC cells for 96 h. Then, we collected the culture medium and performed ELISA. We measured the levels of SDF-1, EGF, bFGF, and IL-6 and found that only SDF-1 in HT-29 cells and CCD-18Co co-culture medium was significantly increased (**P<0.01); there were little changes in expression of the other three cytokines (Figure 5A,B). To determine if integrin αvβ6 influences the expression of SDF-1, we collected cell culture media from the above-mentioned CRC cells after they were co-cultured with CCD-18Co for 96 h. The results showed that integrin αvβ6 increased the secretion of SDF-1 by CAFs, which was decreased upon β6 knockdown by siRNA (**P<0.01; Figure 5C,D).

#### CAFs induce CRC progression via the SDF-1/CXCR4 axis

To investigate if the SDF-1/CXCR4 axis was involved in CAF-mediated induction of CRC cell progression, we co-cultured HT-29 or RKO CRC cells with NFs or active CAFs for 24 h, followed by the transwell invasion assay. The number of invasive CRC cells significantly increased when co-cultured with CAFs (**P<0.01). However, when the co-cultured cells were pretreated with AMD3100 (a CXCR4 antagonist), these effects almost disappeared (Figure 5E,F).

## Discussion

Malignant tumors are regarded as the result of an imbalance between tumor cells and normal cells [22], and the TME plays a very important role in this process. The TME is a complex environment for the survival of tumor cells and is composed of multiple proteins and cell types. The TME not only plays a supporting role for various cells, including tumor cells, immune cells, vascular endothelial cells, and fibroblasts but also forms the ECM (including collagen, laminin, and proteoglycan complexes) and secretes multiple cytokines, such as SDF-1 [23], IL-6 [24], bFGF [25], and EGF [26]. The cancer cell can re-construe the TME; moreover, the re-construed TME can also further influence cancer cell behaviors and conditions, which illustrates the cross-talk between cancer cells and the TME [27].

Fibroblasts comprise a large proportion of cells within the TME. When they are recruited to the malignant tumor zone, they become transformed into CAFs, which are sometimes called reactive stromal fibroblasts, myofibroblasts, or activated fibroblasts [28]. CAFs are mainly derived from three kinds of cells. The first type is the stromal fibroblast, which is thought to be the primary source of CAFs via the process of transdifferentiation of fibroblasts to myofibroblasts [29]. Multiple growth factors secreted by cancer cells induce the transformation of fibroblasts during this process [30,31]. Second, some cancer cells can be transformed into MSCs during the EMT, which can further differentiate into CAFs [32]. Third, some CAFS are derived from endothelial cells via the endothelial–mesenchymal transition [33]. A small proportion of CAFs are also derived from other cell types.

FAP is one of the important marker proteins of activated CAFs. In normal NFs, FAP expression can hardly be detected, but its expression increases when the fibroblasts are activated [34]. α-SMA is another marker of activated fibroblasts [35], and α-SMA-positive myofibroblasts are the main constituents of the cell matrix. CAFs play a very important role in regulating the progression of cancer. When they make contact with different tumors, they secrete multiple cytokines that promote tumor cell proliferation. Matsumoto and Nakamura [36] found that activated CAFs can release the hepatocyte growth factor, which mediates tumor–stromal interactions. Wang et al. [37] found that stromal fibroblasts can produce EGF in lung cancer and combine with the EGFR in lung cancer cells to induce drug resistance. Yu et al. [38] found that stromal fibroblasts can be transformed into CAFs by paracrine TGF-β signaling, leading to the induction of breast cancer cell proliferation; our data are in accordance with these results. SDF-1 also called CXCL-12, is the ligand of CXCR4, both of which belong to the CXC chemokine family [39]. Studies from our group and others found that the SDF-1/CXCR4 axis plays a significant role in the progression and metastasis of CRC cells [40,41]. Orimo et al. [42] confirmed that after CAFs are activated in human breast cancer, they secrete SDF-1 to promote cancer cell progression via the SDF-1/CXCR4 axis.

In our study, we found that CRC cells induce a morphology change in fibroblasts isolated in the TME, similar to myofibroblasts (data not shown). Compared with negative integrin αvβ6 expression CRC cells, positive integrin αvβ6 expression in CRC cells accelerate this process. Activated fibroblasts expressed proteins such as α-SMA and FAP, which are typical molecular marker of CAFs. Further investigations are needed to determine the underlying mechanisms. All types of CRC cells in the TME can secrete TGF-β-LAP. Although TGF-β with LAP is an inactive form, integrin αvβ6 can activate TGF-β by degrading LAP. Activated TGF-β can further induce fibroblast activation; subsequently, activated CAFs can induce CRC cell invasion. However, this induction effect was strengthened when CRC cells expressed integrin αvβ6, and it could be inhibited by the CXCR4 antagonist AMD3100. Hence, these data suggest that CAFs induce colon cancer progression via the SDF-1/CXCR4 axis.

Overall, the results of the present study showed that integrin αvβ6 plays a role in the bi-directional regulation of CRC cells and CAFs. Thus, drugs targetting integrin αvβ6 may be potential therapeutic targets for epithelial cell cancers expressing integrin αvβ6.

## Abbreviations

α-SMA: α-smooth muscle actin
bFGF: basic fibroblast growth factor
CAF: cancer-associated fibroblast
CRC: colorectal cancer
CXCR4: C–X–C chemokine receptor type 4
ECM: extracellular matrix
EGF: epidermal growth factor
EMT: epithelial–mesenchymal transition
FAP: fibroblast-activating protein
IL-6: interleukin 6
LAP: latency-associated peptide
MMP: matrix metalloproteinase
MSC: mesenchymal stem cell
NF: normal fibroblast
SDF-1: stromal cell-derived factor-1
TGF-β: transforming growth factor β
TME: tumor microenvironment
